# CO_2_ to CO Electroreduction, Electrocatalytic H_2_ Evolution, and Catalytic Degradation of Organic Dyes Using a Co(II) *meso*-Tetraarylporphyrin †

**DOI:** 10.3390/molecules27051705

**Published:** 2022-03-05

**Authors:** Mouhieddinne Guergueb, Frédérique Loiseau, Florian Molton, Habib Nasri, Axel Klein

**Affiliations:** 1 Faculty of Sciences of Monastir, University of Monastir, Avenue de l’Environnement, Monastir 5019, Tunisia; mhabib.nasri@fsm.rnu.tn; 2 Département de Chimie Moléculaire (DCM), CNRS UMR 5250, Université Grenoble Alpes, F-38000 Grenoble, France; frederique.loiseau@univ-grenoble-alpes.fr (F.L.); florian.molton@univ-grenoble-alpes.fr (F.M.); 3 Department of Chemistry, Faculty of Mathematics and Natural Sciences, Institute for Inorganic Chemistry, University of Cologne, 50939 Cologne, Germany

**Keywords:** cobalt(II) porphyrins, cyclic voltammetry, electrocatalytic hydrogen evolution, electroreduction CO_2_ to CO, catalytic degradation of dyes

## Abstract

The *meso*-tetrakis(4-(trifluoromethyl)phenyl)porphyrinato cobalt(II) complex [Co(TMFPP)] was synthesised in 93% yield. The compound was studied by ^1^H NMR, UV-visible absorption, and photoluminescence spectroscopy. The optical band gap *E*g was calculated to 2.15 eV using the Tauc plot method and a semiconducting character is suggested. Cyclic voltammetry showed two fully reversible reduction waves at *E*_1/2_ = −0.91 V and *E*_1/2_ = −2.05 V vs. SCE and reversible oxidations at 0.30 V and 0.98 V representing both metal-centred (Co(0)/Co(I)/Co(II)/Co(III)) and porphyrin-centred (Por^2−^/Por^−^) processes. [Co(TMFPP)] is a very active catalyst for the electrochemical formation of H_2_ from DMF/acetic acid, with a Faradaic Efficiency (FE) of 85%, and also catalysed the reduction of CO_2_ to CO with a FE of 90%. Moreover, the two triarylmethane dyes crystal violet and malachite green were decomposed using H_2_O_2_ and [Co(TMFPP)] as catalyst with an efficiency of more than 85% in one batch.

## 1. Introduction

Inspired by nature that is using metalloporphyrins as antennae molecules, redox shuttles and redox and photo catalysts [1], researchers have tried to use artificial porphyrin complexes for various purposes in the last decades [2,3,4,5,6,7,8]. Cobalt porphyrins provide a rich electrochemistry consisting of both metal- (Co(I)/Co(II)/Co(III) and porphyrin-centred redox processes. By variation of the substituents on the phenyl core of the porphyrin ligands, these processes can cover a huge range of potentials [9,10,11,12,13,14,15,16,17,18,19,20,21,22,23,24,25]. Moreover, there are one or two axial positions available for binding small molecules [10,11,12,26,27,28,29]. Both properties make Co porphyrins very suitable for redox catalysis [22,23,30,31,32,33,34,35,36,37,38,39,40,41,42,43,44].

In view of the climate crisis, the photochemical or electrochemical conversion of CO_2_ and H_2_O into energy-rich fuel products such as methanol [30,32,33] or dihydrogen (H_2_) [45,46,47] are important goals, and cobalt porphyrin complexes have been studied as catalysts for the CO_2_ reduction [30,33,34,35,36,37,38,39,40,44] and the H_2_ evolution [18,41,42,48], besides other important redox processes such as O_2_ reduction [19,23,49,50,51,52,53,54], O_2_ evolution reaction (OER) [49,55], and interesting organic redox transformations [56,57,58,59,60].

The potential of the CO_2_ reduction is strongly connected to the presence of protons [7,40]. However, fine-tuning of the reduction potentials of Co porphyrins is easily possible through substitution on the ligand core. The workhorses among the porphyrin ligands, the 5,10,15,20-tetraphenyl-porphyrins or *meso*-tetrakis(phenyl)porphyrins (TPP) have been varied in this respect to a large extent at the phenyl and pyrrole positions and these Co(TPP) complexes show reduction potentials in the range of −0.5 to −1.5 V vs. the SCE (saturated calomel electrode) [17,18,23,24,25,30,32,40,44,61,62]. The NHE potentials are converted into the current SCE scale by subtracting about 240 mV, while SCE differs from the ferrocene/ferrocenium couple by +160 mV [63]. As an example, extension of the π-conjugation in the Co(TPP) system, generating the Co(II)(*meso*-tetrakis(4-(pyren-1-yl)phenyl)porphyrin), allowed to move the reduction potential to higher values (easier reduction), which allowed the operation at −0.6 V, a high Faradaic efficiency (FE) for CO production with a high turnover frequency of 2.1 s^−1^ [32]. Co(II)(TPP) complexes in composite materials have also been used for CO_2_ reduction [34,37,64].

Electrochemical hydrogen production suffers from a huge overpotential of the so-called hydrogen evolution reaction (HER) for many materials, and elemental platinum was the first choice for a long time [45,46,47]. In the quest to replace this expensive metal and create molecular redox catalysts allowing the operation of cheaper electrode materials, complexes of abundant transition metals [47,65,66,67,68] containing various ligands including Co porphyrins have been synthesised [18,41,45,47,48,56,66,67,68,69,70]. For example, in a benchmarking work, the so-called hangman porphyrin which contains a proton-transferring COOH group in close proximity to the Co centre allowed very efficient HER catalysis with PhCOOH as substrate at a proton transfer (PT) rate of 3.10^−6^ s^−1^ and an electron transfer (ET) rate constant of 8.5 10^−6^ s^−1^ [69]. In a very early study using Co porphyrins containing *meso*-tetrakis(*N,N,N*-trimethylanilinium-4-yl)porphine chloride, *meso*-tetrapyrid-4-yl-porphine, and *meso*-tetrakis(*N*-methylpyridinium-4-yl)porphine chloride, H_2_ production from aqueous trifluoroacetic acid (TFA) on a Hg pool electrode at −0.95 V reached almost 100% FE [70]. In the same work, the Co(I) species was identified to react very rapidly with protons: Co(I) +H^+^ ⇆ Co(II) + ½ H_2_, while the formation of H_2_ from Co(III)-H^−^ intermediates is slower.

Amongst other organic redox transformations, the electrochemically or photochemically initiated decomposition of organic dyes in waste water is another increasingly important research field in recent years [5]. Recently, Zn(II) [12,15,71,72] and Co(II) porphyrins [10,11,26,73] have been used as catalysts in the degradation of organic dyes with H_2_O_2_. In view of the first oxidation potential of Co(TPP) complexes lying in the range of 0.1 to 0.75 V vs. SCE [10,11,13,16,17,22,24,25,26,61,62], their high reactivity in radical-based organic transformations [14,58,59,60] is not unexpected and a very recent study on a (5,10,15,20-tetrakis(2,5-dimethoxyphenyl)porphyrinato)cobalt(II) has revealed some mechanistic details in such decomposition reactions using H_2_O_2_ [73].

**Scheme 1:**
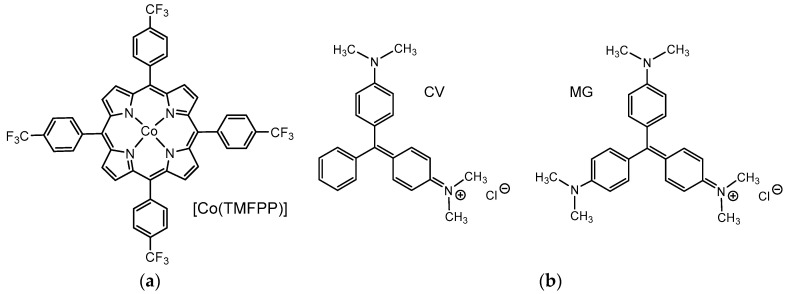
Structures of (**a**) [Co(TMFPP)], (**b**) crystal violet (CV) and malachite green (MG).

We recently stepped on the Co(II) complex [Co(TMFPP)] (H_2_TMFPP = *meso*-tetrakis(4-(trifluoromethyl)phenyl)porphyrin) which has previously been reported [21,29,53,54,59,60,74,75], for example, as catalyst for the direct C–H arylation of benzene [60]. This motivated us to study its use as redox catalyst for the electrocatalytic hydrogen evolution, the electroreduction of CO_2_ to CO, and the catalytic degradation and adsorption of the dyes crystal violet (CV) and malachite green (MG; Scheme 1). We synthesised the compound in a slightly modified procedure and characterised it by elemental analysis, ^1^H NMR, FT-IR, absorption and photoluminescence spectroscopy. We briefly report on basic spectroscopic and electrochemical properties, as this has not been done before, and then report in detail on the electrocatalytical experiments.

## 2. Results and Discussion

### 2.1. Synthesis and ^1^H NMR Spectroscopy

**Figure 1:**
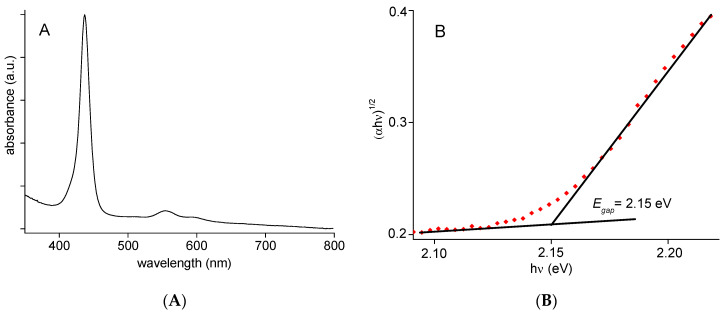
UV/vis absorption spectrum of [Co(TMFPP)] in CH_2_Cl_2_ (**A**); curve of (αhυ)^2^ as a function of photon energy *E* (**B**).

The free-base porphyrin *meso*-tetrakis(4-(trifluoromethyl)phenyl)porphyrin (H_2_TMFPP) was synthesised modifying a literature method [76] (see Section 3). Elemental analysis and ^1^H NMR confirmed the purity of the material. The *meso*-tetrakis(4-(trifluoromethyl)phenyl)porphyrinato Co(II) [Co(TMFPP)] (Scheme 1) was synthesised using the so-called DMF method [77]. Elemental analysis and MS confirmed its purity. FT-IR (Figure S1, Supplementary Material) and UV-vis absorption (Figure 1) and ^1^H NMR data (Figure S2) agree with the reported data [51]. In keeping with the paramagnetic character of the Co(II) d^7^ system, we obtained broadened ^1^H NMR signals at *δ* = 15.71 ppm for the β-pyrrole protons and at 12.96 and 9.92 ppm for the phenyl atoms [10,11,26,54,78].

### 2.2. Photophysical Properties

The UV-vis absorption spectrum of [Co(TMFPP)] in CH_2_Cl_2_ solution showed a Soret band at 437 nm and a Q band at 554 nm in line with similar Co(II) porphyrins (Figure 1A) [51,53,79,80,81,82]. The optical band gap (*E*g), which is the energy difference between the HOMO and LUMO levels, was determined from the UV-vis absorption spectrum. The values of the optical band gap (*E*g) of [Co(TMFPP)] were determined using the Tauc relation (Figure 1B). The *E*g value was 2.15 eV, which is in the normal range for Co metalloporphyrins [53,80].

**Figure 2:**
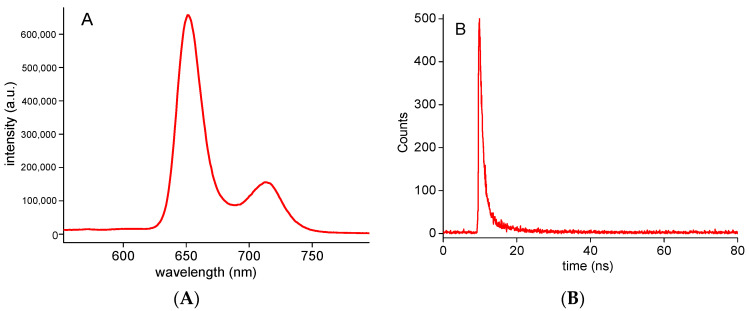
Emission spectrum of [Co(TMFPP)] in 10^−6^ M solutions in CH_2_Cl_2_ at room temperature (**A**), excited at 405 nm; fluorescence decay profile (**B**).

The photoluminescence spectrum of [Co(TMFPP)] in CH_2_Cl_2_ at room temperature is shown in Figure 2. Upon excitation at 405 nm, an emission with maxima at 653 and 718 nm is observed and can be attributed to the *S*_1_[Q(0,0)]→*S*_0_ and *S*_1_[Q(0,1)]→*S*_0_ transitions in line with previous studies on [Co(TPP)] [80,83,84,85] and related [Zn(TPP)] [12,15,72,81]. The photoluminescence quantum yield (Φ_PL_) for [Co(TMFPP)] is 0.041. The singlet excited-state lifetimes were measured by the single-photon counting technique, and the fluorescence decays were fitted to simple exponentials with 2 ns lifetime (Figure 2B), which lies in a typical range for [Co(TPP)] derivatives [80,83].

### 2.3. Electrochemical Characterisation

**Figure 3:**
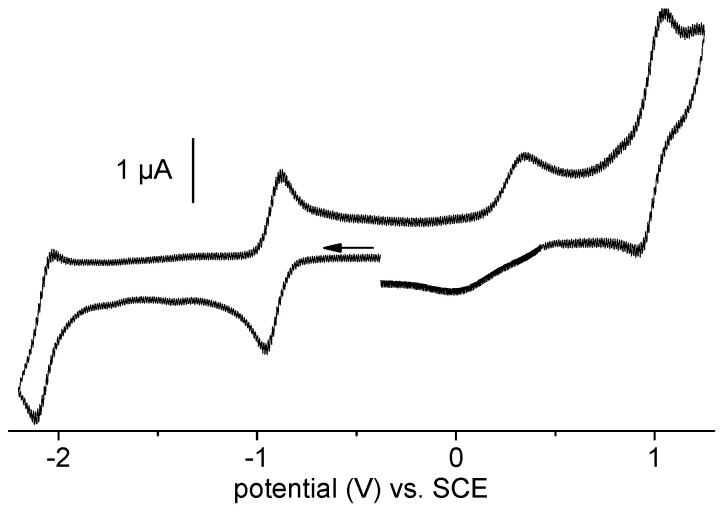
Cyclic voltammogram of [Co(TMFPP)] in 0.1 M *n*Bu_4_NBF_4_/DMF, recorded at 100 mV s^−1^.

Cyclic voltammograms of [Co(TMFPP)] were recorded in DMF, which is a potential donor ligand and is thus prone to coordinate to the Co centre after oxidation, as has been found for most square planar coordinated M(II) porphyrins [58,59]. Two reversible one-electron reductions were found for [Co(TMFPP)] at *E*_1/2_ = −0.91 V and *E*_1/2_ = −2.05 V (Figure 3). While it is generally accepted to assign the first wave to the Co(II)/Co(I) redox couple [24,61,62,86,87], the second was earlier discussed as porphyrin-centred (Por^2−^/Por^3−^), in line with reports on the unsubstituted [Co(TPP)] [24,61,62,86]. Alternatively, a Co(0) species after a Co(I)/Co(0) reduction has been discussed [20,88,89]. The latter description is supported by UV-vis absorption spectroscopy [89].

A first one-electron oxidation at 0.30 V that can be assigned to the Co(II)/Co(III) redox couple is broadened, but reversible. The broadening is due to the coordination of DMF after oxidation, as mentioned above. This wave is followed by a slightly larger reversible wave at 0.98 V, which is assigned to a porphyrin-centred process (Por^2−^/Por^1−^) in line with previous reports [21,24,61,62,85,86,87]. For the 4-MeO substituted derivative, a third oxidation at 1.09 V following the second oxidation at 0.92 V was reported [24,62], and for the 2,5-MeO substituted complex, second and third oxidation waves were observed at 0.62 and 1.15 V [73]. This lets us assume that, for [Co(TMFPP)], both these two porphyrin-centred processes are merged into one (larger) wave.

For [Co(TPP)] in DMF potentials of −1.88, −0.77, 0.30, and 1.05 V were previously reported for the same processes [24], while the 4-MeO substituted derivative showed −0.98 V, 0.38 V, and 0.92 V for the first reduction and the two oxidations. This means that the introduction of the four CF_3_ groups does not markedly affect the metal-centred first oxidation and reduction.

**Figure 4:**
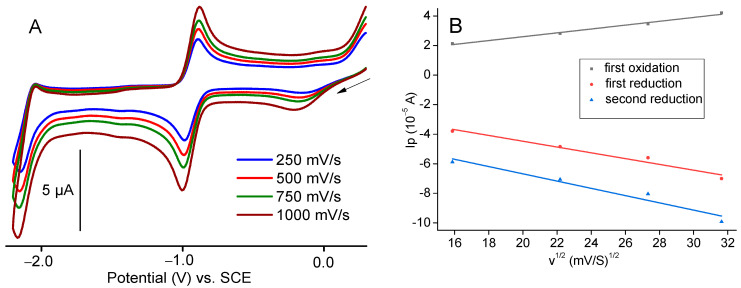
Cyclic voltammograms of [Co(TMFPP)] in 0.1 M *n*Bu_4_NBF_4_/DMF recorded at different scan rates (**A**). Peak currents vs. square roots of the scan rate for the two reduction processes (**B**).

For a fully homogeneous diffusion-controlled electrochemical process, the peak current (*I*_p_) for a Faradaic electron transfer varies linearly with the square root of scan rate (ν^1/2^). From the slope of the *I*_p_ vs. ν^1/2^ plot, the diffusion coefficient (*D*) can be determined using Randles–Sevcik equation (Equation (1)):

*I*_p_ = 0.4463 F *A* (F/R*T*)^1/2^ *D*^1/2^ n_p_^3/2^ [*C*_0_] ν^1/2^ (1)

where *I*_p_ is the peak current, F is the Faraday constant (F = 96485 C mol^−1^), R is the universal gas constant (R = 8.314 J K^−1^ mol^−1^), *T* = 298 K, n_p_ is the number of electrons transferred (here, n_p_ = 1), *A* is the active surface area of the electrode (0.00785 cm^2^). Note that our plots are reported as a function of the current density, bypassing the need of the area value in Equation (1). *D* is the diffusion coefficient for the complex, [*C*_0_] is the concentration of the catalyst (here [*C*_0_] = 1 mM), and ν is the scan rate in V s^−1^. The diffusion coefficient (*D*) was calculated from the slope of *I*_p_ vs. ν^1/2^ (Figure 4, right). The diffusion coefficient *D* for the Co(II)/Co(I) reduction is 1.98 × 10^−7^ cm S^−1^, while the value for the Co(II)/Co(III) oxidation is slightly larger with 9.5 × 10^−7^ cm S^−1^, in keeping with the assumed additional DMF ligand for the oxidised complex [Co(TMFPP)(DMF)]^+^. The diffusion coefficient *D* for the second reduction process with 1.1 × 10^−8^ cm S^−1^ is smaller than the value for the first reduction, which is due to the increased negative charge, but still quite large.

### 2.4. Electrocatalytic H_2_ Production in the Presence of Acetic Acid (AcOH)

**Figure 5:**
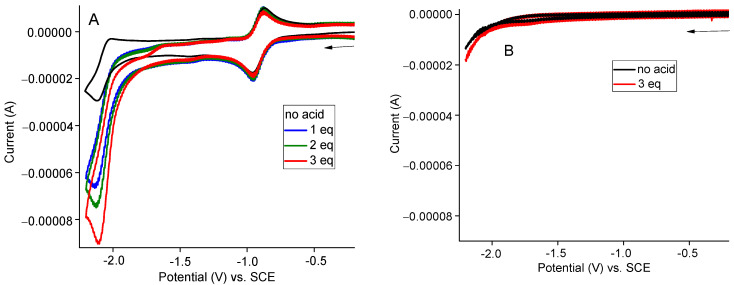
Cyclic voltammograms of [Co(TMFPP)] (1 mM) in 0.1 M *n*Bu_4_NBF_4_/DMF recorded in the absence (black trace) or in the presence of 1 to 3 eq. CH_3_COOH in DMF at 100 mV s^−1^ (**A**), Blank test without catalyst (**B**).

We studied the electrocatalytic activity of [Co(TMFPP)] in DMF/acetic acid due to better solubility in this mixture than in water (Figure 5).

On first view, our cyclic voltammetric plots show that, upon addition of acid, a catalytic current appears at the second reduction wave of [Co(TMFPP)] (Figure 5), while the first wave remains unchanged. The FE in H_2_ production (quantified by GC) of [Co(TMFPP)] at −2.3 V was determined after 2 h to 85%. For the previously reported [Co(TMAP)](ClO_4_)_2_ (H_2_TMAP = *meso*-tetrakis(*N,N,N*-trimethylanilinium-4-yl)porphine), [Co(TMPyP)](ClO_4_)_2_ (*meso*-tetrakis(*N*-methylpyridinium-4-yl)porphine) and [Co(TpyP)] (*meso*-tetrapyrid-4-ylporphine), FEs of >90% were found [70].

Mechanistically speaking, it seems that the first reduced species which we formally describe as [Co(I)(Por^2−^]^−^ is not catalytically very active, which would stand in contrast to previous mechanistic studies [18,70] which proposed that the reduced Co(I) species reacts with protons forming Co(II) and Co(III) species (Equations (2) to (6)) [70].

Co(I) +H^+^ ⇆ Co(II) + ½ H_2_ (2)

Co(II) +e^−^ ⇆ Co(I) (3)

Co(I) +H^+^ ⇆ Co(III)H^−^ (4)

Co(III)H^−^ +H^+^ ⇆ H_2_ + Co(III) (5)

Co(III)H^−^ +H^+^ ⇆ ½ H_2_ + Co(II) (6)

Our experiments indicate that only after the second reduction, the resulting [Co(0)(Por^2−^)]^2−^ species is active in reducing protons. In the abovementioned study on the complexes [Co(TMAP)](ClO_4_)_2_, [Co(TMPyP)](ClO_4_)_2_, and [Co(TpyP)] catalytic currents representing the proton reduction were observed at −0.95 V [70]. In a recent study, very different behaviour was found for the catalytic proton reduction using [Co(TXPP)] (H_2_TXPP = *meso*-tetra-*para*-X-phenylporphin) catalysts [18]. For X = Cl = catalytic waves were observed at around −2 V comparable to our findings, while for X = OMe, the H_2_ evolution was observed already at around −1 V. Thus, we can conclude that the substitution pattern of the *meso*-tetraarylporphin ligands has a strong impact on the observed catalytic potential. Depending on these patterns, the Co(TPP) derivatives might be an active catalyst in the Co(I) oxidation state or alternatively might need to reach the Co(0) state after the second reduction for efficient proton reduction.

### 2.5. Electroreduction CO_2_ to CO

**Figure 6:**
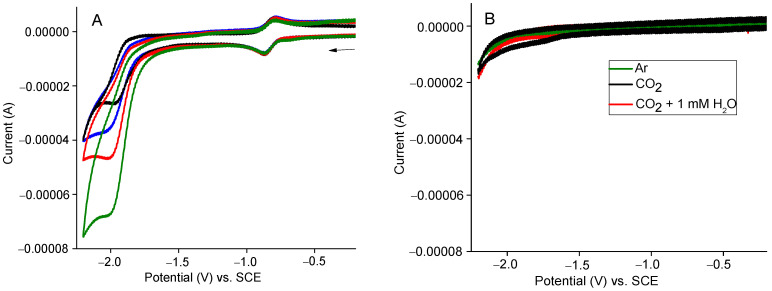
Cyclic voltammograms of 1 mM solutions of [Co(TMFPP)] in 0.1 M *n*Bu_4_NBF_4_/DMF in the presence of H_2_O (Black: in Ar, Red: in CO_2_, Blue: in Ar and 1 mM of H_2_O and green: in CO_2_ and 1mM of H_2_O) (**A**). Blank test without catalyst (**B**).

[Co(TMFPP)] was further tested for the electrocatalytic CO_2_ reduction, in CO_2_-saturated DMF, with water added as a proton source. Cyclic voltammograms of [Co(TMFPP)] in the presence of CO_2_ showed marked catalytic currents at potentials at around −2 V, with the presence of water being beneficial (Figure 6A, green and red trace). No activity was found in the absence of the catalyst (Figure 6B).

Controlled potential electrolysis at −2.25 V for 2 h in aqueous DMF under a CO_2_ atmosphere gave a FE of 90%; GC confirmed the production of CO and only traces of H_2_. Remarkably, not even traces of the very common products formate and methanol were found.

[Co(TPP)] was reported with an FE of 50% for CO alongside with traces of H_2_ (FE = 2%), formate (4%), acetate (2%) and oxalate (0.4%) on electrolysis at −1.95 or −2.05 V vs. SEC in DMF [88]. When immobilised on carbon nanotubes, [Co(TPP)] allowed an efficiency of 83% at −1.15 V or 93% at −1.35 V [88] and the authors could circumvent the rapid catalyst decomposition monitored by UV-vis absorption spectroscopy.

Thus, our unsupported [Co(TMFPP)] is markedly superior in terms of efficiency and selectivity to the standard [Co(TPP)] and support with an electron-conducting material might pave the way to operate the [Co(TMFPP)] at less negative potentials. Importantly, also here, only the second reduction wave produces the catalytically active species, which we describe as Co(0) complex.

### 2.6. Catalytic Oxidative Degradation of Dyes

**Figure 7:**
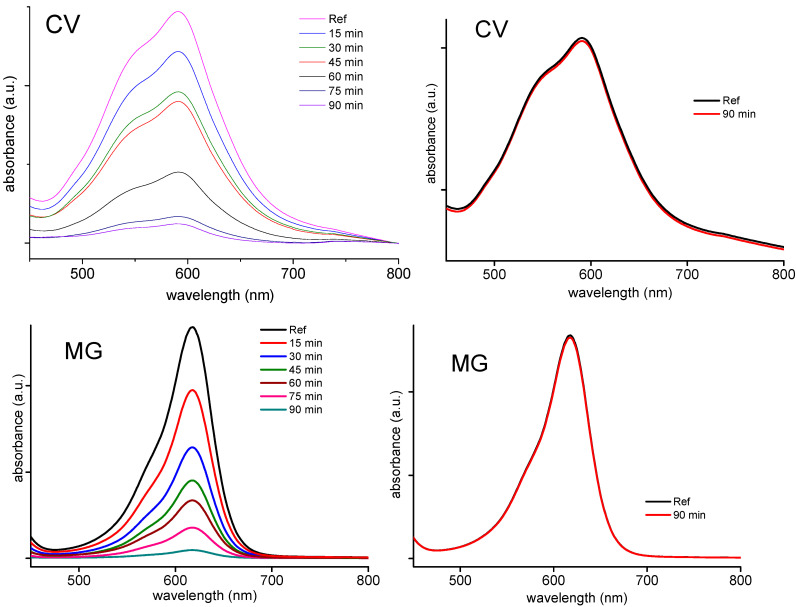
Evolution of the absorbance of malachite green (MG) and crystal violet (CV) over time using 3 mL H_2_O_2_ and 5 mg [Co(TMFPP)] and dye concentrations of 25 mg L^−1^, in H_2_O at pH = 8 and *T* = 298 K (**left**). Blank tests without catalyst (**right**).

To further evaluate the catalytic properties of [Co(TMFPP)], we studied the decomposition of the two dyes malachite green (MG, 4-([4-(dimethylamino)phenyl](phenyl)methylidene)-*N,N*-dimethylcyclohexa-2,5-dien-1-iminium chloride) and crystal violet (CV, 4-(bis[4-(dimethylamino)phenyl]methylidene)-*N,N*-dimethylcyclohexa-2,5-dien-1-iminium chloride; see Scheme 1) in the presence of H_2_O_2_. The MG cation shows a very intense green colour with an absorption band centred at 621 nm, while the CV cation has a very intense violet colour and the absorption maximum of the most intense band at 591 nm (Figure 7). Upon addition of H_2_O_2_ in the presence of [Co(TMFPP)], the dyes were rapidly decomposed, as the UV-vis absorption traces showed (Figure 7).

To further study the reaction kinetics of the degradation, the *C*_t_/*C*_0_ ratios were varied and a pseudo-first order rate constant *k* was calculated using Equation (7) (Langmuir–Hinshelwood):

ln *C*_0_/*C*_t_ = *k t* (7)

**Figure 8:**
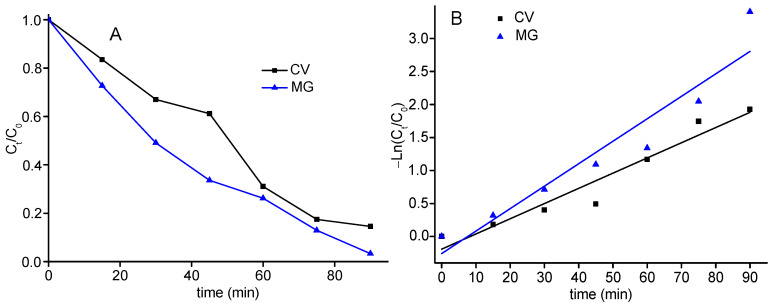
Kinetics of [Co(TMFPP)]-catalysed degradation of malachite green (MG), and crystal violet (CV) through H_2_O_2_ in aqueous solution: changes in *C*_t_/*C*_0_ versus time (**A**) and changes in ln(*C*_t_/*C*_0_) versus time (**B**).

*C*_t_ and *C*_0_ are the dye concentrations at times *t* and 0, *k* is the first-order rate constant. The fit of the pseudo kinetic model is shown in Figure 8, and the rate constants of degradation (*k*) were calculated to 0.023 and 0.034 min^−1^ for CV and MG, respectively.

The two triarylmethane dyes crystal violet and malachite green were decomposed using H_2_O_2_ and [Co(TMFPP)] as catalyst with an efficiency of more than 85% (*C*_0_ = 25 mg/L, pH = 8, H_2_O_2_ concentration = 3 mL/L, *T* = 25 °C). Two 4-cyanopyridine complexes of the type [Co(II)(Por)(4-CNpy)] (Por = *meso*-tetrakis(*para*-methoxyphenyl)porphyrinato and *meso*-tetra(*para*-chlorophenyl)porphyrin) as catalysts in the degradation of organic dyes using H_2_O_2_ gave a degradation efficiency of more than 78% [10,11,26,73], while recently reported Zn(II) triazole-substituted *meso*-arylsubstituted porphyrin complexes gave efficiencies of up to 50% [12,15,71,72]. This shows that Co(II) porphyrins are generally superior to Zn(II) derivatives in line with the assumption that the first reduction is cobalt-centred (Co(II)(Co(I)).

## 3. Experimental Section

### 3.1. Materials

All reagents and solvents were purchased from ACROS ORGANICS (Geel, Belgium) or Sigma Aldrich (St. Louis, MO, USA). Solvents were purified using literature methods [90]. Silica gel 150 (35–70 μm particle size, Davisil) was used for final purification of the products. Double-distilled water was used in the experiments.

### 3.2. Synthetic Procedures

#### 3.2.1. Synthesis of the *meso*-Tetrakis(4-(trifluoromethyl)phenyl)porphyrin (H_2_TMFPP)

Note that 3.65 g of 4-(trifluoromethyl)benzaldehyde (21 mmol) were dissolved in propionic acid (100 mL) in air and heated to 120 °C. In addition, 1.4 g of pyrrole (1.35 mL, 21 mmol) were added dropwise to the reaction and the mixture was kept at 120 °C for a further 45 min. The resulting solution was allowed to cool and the tarry mixture was filtered to give a black solid, which was rinsed with water (5 × 100 mL) and (5 × 100 mL) *n*-hexane and finally dried under vacuum with a yield of 1.25 g (1.4 mmol, 27%). Anal. calcd. for C_48_H_26_F_12_N_4_ (886.74): C, 65.02; H, 2.96; N, 6.32; found: C, 65.21; H, 2.85; N, 6.43%; MS (ESI(+), CH_2_Cl_2_): m/z = 886.68 for [M]^+^; UV-vis (CH_2_Cl_2_): λ_max_ (ε.10^−3^M^−1^cm^−1^): 424(370), 519(85), 557(39), 591(30), 651(25); ^1^H NMR (500 MHz, CDCl_3_) δ = 8.95 (s, 8H, β-pyrrole), 8.13 (s, 8H, arylH), 7.77 (s, 8H, arylH) ppm. FT-IR (solid, $\bar{\nu }$, cm^−^^1^); 3290 (νNH), 2922 (νCH), 1518 (νC=N/νC=C), 965 (δCCH).

#### 3.2.2. Synthesis of the *meso*-Tetrakis(4-(trifluoromethyl)phenyl)porphyrinato Co(II) [Co(TMFPP)]

An amount of 200 mg (0.225 mmol) H_2_TMFPP was dissolved in 100 mL of DMF. The solution was brought to reflux under magnetic stirring. After dissolution of H_2_TMFPP or H_2_TTMPP, 53 mg (0.222 mmol) CoCl_2_.6H_2_O were added. The reaction mixture was left under stirring for 3 h. Thin-layer chromatography (Al_2_O_3_, with CH_2_Cl_2_ as eluent) showed no free-base porphyrin- at this level. After this, the solution was brought to 45–55 °C, and 100 mL H_2_O were poured in. The resulting solid was filtered, washed with *n*-hexane and finally dried under vacuum to yield 195 mg (206 mmol, 93%) of product. Anal. calcd. for C_48_H_24_N_4_F_12_Co (943.66): C, 61.09; H, 2.56; N, 5.94; found: C, 60.99; H, 2.59; N, 6.02%; MS (ESI(+), CH_2_Cl_2_): m/z = 943.62 for [M]^+^; UV-vis (CH_2_Cl_2_): λ_max_ (ε.10^−3^M^−1^cm^−1^): 414(380), 533(54), 568 sh(16), 437(385), 554(30), 592(20); ^1^H NMR (500 MHz, CDCl_3_): δ = 15.71 (s, 8H, β-pyrrole); 12.96 (s, 8H, arylH); 9.92 (s, 8H, arylH); FT-IR (solid, $\bar{\nu }$, cm^−^^1^); 2959 (νCH porphyrin), 1498 (νC=N/(νC=C porphyrin)*,* 1021 (δCCH porphyrin).

### 3.3. Instrumentation

UV-vis absorption spectra were recorded on a WinASPECT PLUS (validation for SPECORD PLUS version 4.2, WinASPECT, Jena, Germany) scanning spectrophotometer using 10 mm path length cuvettes. ^1^H NMR spectra were measured on Bruker DPX 500 spectrometers (Bruker, Rheinhausen, Germany) in CDCl_3_ with the solvent peak as an internal standard. FT-IR spectra were measured on a Perkin Elmer Spectrum Two FT-IR spectrometer (Perkin Elmer, Darmstadt, Germany). Elemental analysis and mass spectrometry were carried out in the nanobio chemistry platform of the ICMG, Grenoble, France. A Fluoromax-4 spectrofluorometer (Horiba Scientific, 59120 Loos, France) was used to record photoluminescence (PL) spectra at room temperature in CH_2_Cl_2_. PL quantum yield (Φ_PL_) was determined using the optical method [12] with [Zn(TPP)] as standard (Φ_PL_ = 0.031). The luminescence lifetime detection was performed upon irradiation at λ = 405 nm. The luminescence decay was analysed using the PicoQuant FLUOFIT software (PicoQuant, Berlin, Germany) [15].

### 3.4. Electrochemistry

Cyclic voltammetry experiments were performed using a CH-660B potentiostat at room temperature. All measurements were performed in DMF with a solute concentration of approximately 10^−3^ M and *n*Bu_4_NBF_4_ (0.1 M) as supporting electrolyte. A three-electrode cell was set up with a glassy carbon working electrode, a Pt wire as counter electrode, and an Ag/AgNO_3_ reference electrode. Potentials were converted into values for the saturated calomel electrode (SCE) by applying Equation (8) [11,63,91,92]:

*E*(SCE) = *E*(Ag/AgNO_3_) + 360 mV (8)

### 3.5. Electrocatalytic CO_2_ Reduction

The experiments were performed at room temperature under a CO_2_ atmosphere in a conventional three-electrode cell sealed with Apiezon M vacuum grease. A glassy carbon electrode plate (2 cm^2^, 0.25 mm thickness) was used as the working electrode in the cathodic compartment. A 0.5 mm diameter platinum wire (10 cm length) was used as the counter electrode in the anodic compartment. The cell was purged with Ar or CO_2_ for a minimum of 15 min before controlled potential electrolysis was carried out. Constant magnetic stirring was applied during electrolysis.

### 3.6. Gas Detection

Gas analyses were performed using a GC/MS gas chromatography (Perkin Elmer Clarus 560) instrument with a thermal conductivity detector fitted with RT-QPlot pre column + molecular sieve 5Å column. The temperature was held at 150 °C for the detector and 80 °C for the oven. The carrier gas was helium. Manual injections of 100 μL were performed during the experiment via a gas-tight Hamilton microsyringe. The total volume of the cell was 173 mL.

### 3.7. Faradaic Efficiency Calculation

The Faradaic Efficiency (FE) of the CO_2_ reduction or hydrogen evolution reaction (HER) was calculated using Equation (9):

FE = *Z n F*/*Q* (9)

where *Z* is the amount of product in mol, *n* is the number of the electrons (2 for both CO and H_2_), *F* is the Faraday constant, and *Q* is the number of electrons (or charge) passed through the solution during electrolysis (*I t*).

### 3.8. Catalytic Dye Degradation

In a typical investigation, to a 10 mL aqueous solution of the dyes crystal violet (CV) and malachite Green (MG) (20 mg L^−1^), 3 mL/L of H_2_O_2_ (30 wt %) were added. Next, 5 mg of the catalyst were added to this mixture at a stirring speed of 250 rpm. The reaction solution was pipetted into a quartz cell and UV-vis absorption spectra were recorded at different reaction times. Blank experiments were carried out to confirm that the reactions did not take place without catalyst in the presence of H_2_O_2_.

## 4. Conclusions

In this work, the *meso*-tetrakis(4-(trifluoromethyl)phenyl)porphyrinato cobalt(II) complex [Co(TMFPP)] was synthesised via modified literature methods in an excellent yield of 93% from the free-base porphyrin *meso*-tetrakis(4-(trifluoromethyl)phenyl)porphyrin (H_2_TMFPP). Elemental analysis, FT-IR, and ^1^H NMR spectroscopy confirmed the molecular entities. UV-vis absorption spectroscopy localised the Soret band at 437 nm and the Q band at 554 nm. The optical band gap *E*g was calculated using the Tauc plot method to 2.15 eV. The (αhυ)^2^ over *E* plot suggests a semiconducting behaviour of the material. Cyclic voltammetry of the title compound showed two fully reversible reduction waves. Both the first wave, observed at *E*_1/2_ = −0.91 V vs. SCE, and the second at *E*_1/2_ = −2.05 V are ascribed to cobalt-centred processes Co(II)/Co(I) and Co(I)/Co(0), respectively. The first oxidation wave at around 0.3 V is metal-centred Co(II)/Co(III) and broadened through the interaction of the DMF solvent with the oxidised complex. A second oxidation is following at 0.98 V, which is presumably due to the redox couple Por^2−^/Por^−^. [Co(TMFPP)] is a very active catalyst for the electrochemical formation of H_2_ from DMF/acetic acid, with a Faradaic Efficiency (EF) of 85% at a working potential of −2.3 V. This is in line with [Co(0)(Por^2−^)]^2−^ being the active species. The complex also catalysed the reduction of CO_2_ to CO in aqueous DMF under CO_2_ atmosphere with a high EF of 90% and only traces of H_2_ by-product, making our derivative superior to the standard [Co(TPP)]. Also here, catalytic currents are only observed at potentials coinciding with the second reduction potential in the voltammograms, thus the same [Co(0)(Por^2−^)]^2−^ species seems to be active as for the proton reduction. Moreover, the two triarylmethane dyes crystal violet and malachite green were decomposed in aqueous solution using H_2_O_2_ and [Co(TMFPP)] as catalyst with an efficiency of more than 85% in one batch. Given the high stability of the complex and the relatively easy preparation with excellent yields, this makes [Co(TMFPP)] a versatile catalyst for important electrocatalytic reductions and oxidations. The performance on the cathodic side might be improved with the goal of less negative working potentials in future work by blending the complex with electroactive materials such as carbon nanotubes, or by immobilising the complex directly on electrodes.

## Data Availability

Not applicable.

## Supplementary Materials

The following information is available online. Figure S1: FT-IR spectrum of [Co(TMFPP)]. Figure S2: ^1^H NMR spectrum of [Co(TMFPP)] (C ~10^−3^ M) in CDCl_3_.
