# Supplementary material for: CO2 to CO Electroreduction, Electrocatalytic H2 Evolution, and Catalytic Degradation of Organic Dyes Using a Co(II) meso-Tetraarylporphyrin
Source: Molecules. 2022 Mar 5;27(5):1705. doi: 10.3390/molecules27051705 (PMC8912110; doi:10.3390/molecules27051705)
Supplement: Supplementary file 1 [file molecules-27-01705-s001.zip › molecules-1634490-supplementary.pdf]

Supplementary Materials

for

CO_2 to CO Electroreduction, electrocatalytic H_2 evolution, and catalytic degradation of organic dyes using a Co(II) *meso*-tetraarylporphyrin

Mouhieddinne Guergueb [1,*], Frédérique Loiseau [2], Florian Molton [2], Habib Nasri[1], Axel Klein [3,*]

[1] University of Monastir, Faculty of Sciences of Monastir, Avenue de l'Environnement, 5019 Monastir, Tunisia. Dr. Habib Nasri, email: habib.nasri@fsm.rnu.tn, ORCID: 0000-0002-7565-8240 (H.N.)
[2] Département de Chimie Moléculaire (DCM), CNRS UMR 5250, Université Grenoble Alpes, F-38000 Grenoble, France. Prof. Dr. Frederic Loiseau, email: frederique.loiseau@univ-grenoble-alpes.fr, ORCID: 0000-0003-3648-4863 (F.L.); Florian Molton, email: florian.molton@univ-grenoble-alpes.fr; ORCID: 0000-0001-6675-5551 (F.M.)
[3] University of Cologne, Faculty of Mathematics and Natural Sciences, Department of Chemistry, Institute for Inorganic Chemistry, Cologne, Germany

* Corresponding authors: Dr. Mouhieddinne Guergueb, email: mouhieddinneguergueb@gmail.com, ORCID: 0000-0001-7583-3099 (M.G.), Prof. Dr. Axel Klein, email: axel.klein@uni-koeln.de, ORCID: 0000-0003-0093-9619 (A.K.)

contents:

Figure S1. FT-IR spectrum of [Co(TMFPP)].

Figure S2. ^{1}H NMR spectrum of [Co(TMFPP)] (C ~10^{-3} M) in $CDCl_3$ at room temperature.

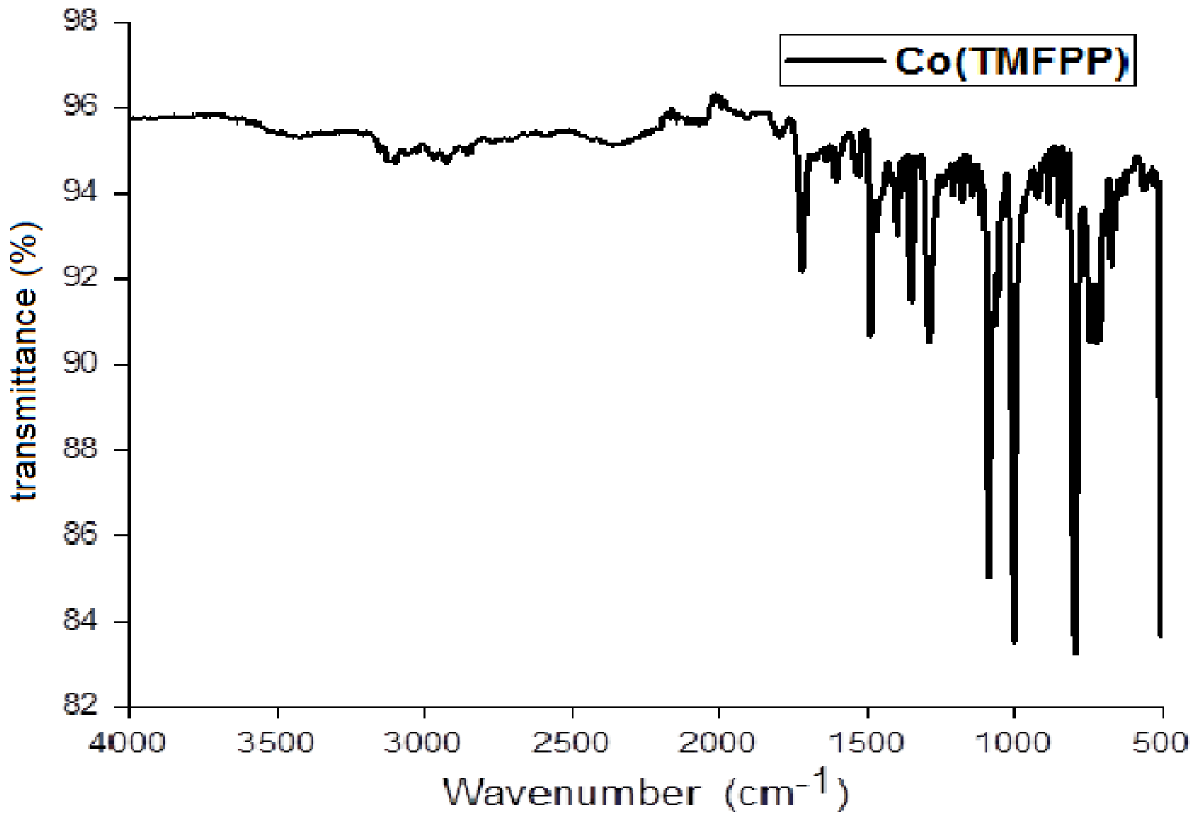

Figure S1. FT-IR spectrum of a solid sample of [Co(TMFPP)].

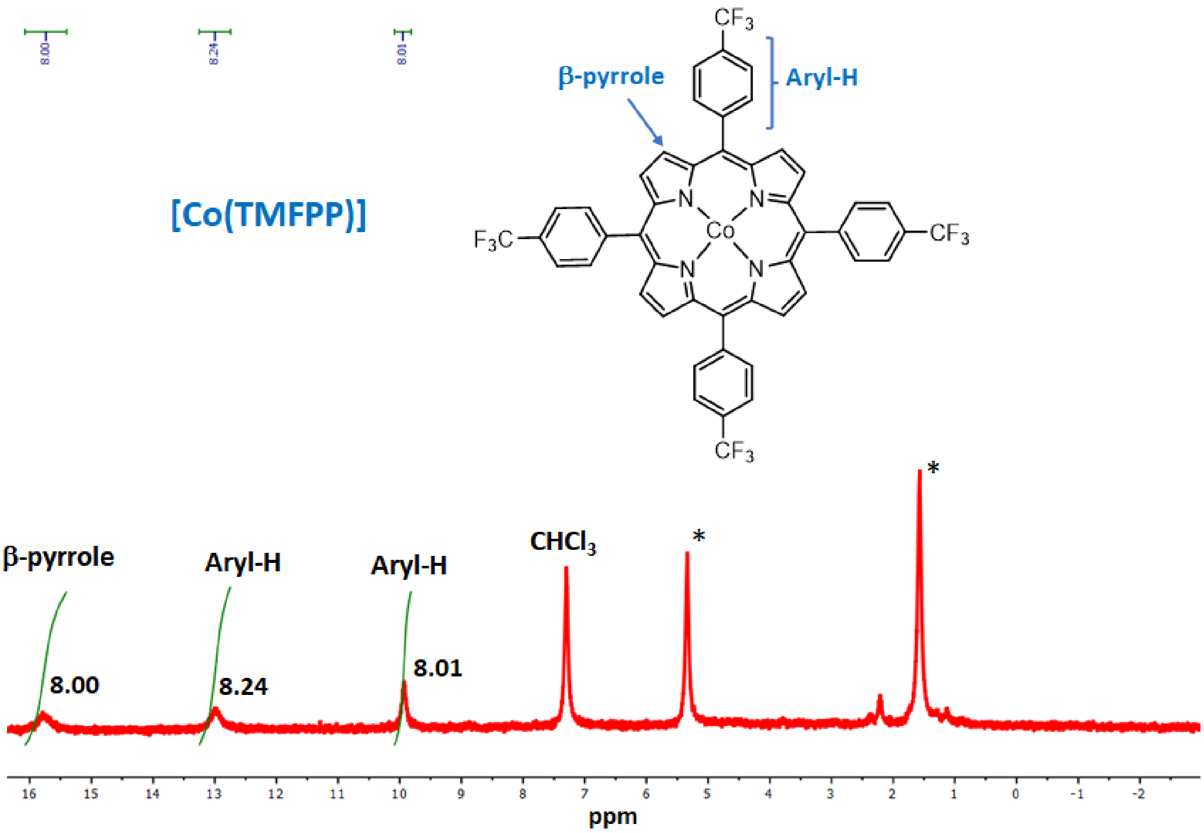

Figure S2. ^{1}H NMR spectrum of [Co(TMFPP)] (C ~10^{-3} M) in $CDCl_3$ at room temperature.
